# Supplementary material for: Impact of sex on use of low tidal volume ventilation in invasively ventilated ICU patients—A mediation analysis using two observational cohorts
Source: PLoS One. 2021 Jul 14;16(7):e0253933. doi: 10.1371/journal.pone.0253933 (PMC8279424; doi:10.1371/journal.pone.0253933)
Supplement: S1 File — (DOCX) [file pone.0253933.s001.docx]

Supporting Information with:

**Impact of sex on use of low tidal volume ventilation in invasively ventilated ICU patients – A mediation analysis using two observational cohorts**

**S1 Supporting Methods**

Page 3. Study design

Page 3. Ethical approval

Page 4. Technical validation

Page 5. Other data preparation and extraction

Page 5. Additional description of the mediation analyses

**S1 Supporting Tables**

Page 6. Table 1. Baseline Characteristics of the Included Patients

Page 8. Table 2. Baseline Characteristics of the Included Patients According to Tertiles of Year of Admission

Page 10. Table 3. Ventilatory Parameters in the First Two days of Ventilation According to Tertiles of Year of Admission

Page 12. Table 4. Effect of Sex on Low Tidal Volume Ventilation According to Tertiles of Year of Admission

**S1 Supporting Figures**

Page 13. Fig 1. Study flow chart

page 14. Fig 2. The trend of height and weight during the years

Page 15. Fig 3. Percentage of patients receiving default tidal volume

Page 16. Fig 4. Absolute tidal volume and corrected tidal volume (by predicted body weight or absolute body weight) over years and according to tertiles of year in patients with sepsis

Page 17. Fig 5. Absolute tidal volume and corrected tidal volume (by predicted body weight or absolute body weight) over years during controlled vs assisted ventilation in the MIMIC-III database

**Supporting References**

Page 18. References

**S1 SUPPORTING METHODS**

**Study design**

The ‘Medical Information Mart for Intensive Care III database’ (MIMIC–III v1.4) is a freely accessible database [1, 2]. It contains administrative data, medication and laboratory data, vital signs, notes and radiology reports from over 50.000 patient admissions between 2001 and 2012 from the Beth Israel Deaconess Medical Center (BIDMC) in Boston, MA. The database is hosted by the Laboratory for Computational Physiology at the Massachusetts Institute of Technology (MIT).

The ‘eICU Collaborative Research Database’ v1.2 contains data from around 200.000 adult patients, who were admitted to one of the 208 participating intensive care units in the United States between 2014 and 2015 [3]. The data was gathered using the tele–ICU services from the Philips eICU program. The Philips eICU Research Institute (eRI) and the Laboratory for Computational Physiology at the MIT, which maintains the data, has generously contributed the ‘eICU Collaborative Research Database’ described here.

**Ethical approval**

The Institutional Review Board of the Beth Israel Deaconess Medical Center (2001–P–001699/14) and the Massachusetts Institute of Technology (No. 0403000206) approved the MIMIC project. Requirement for individual patient consent was waived because the project did not impact clinical care and all protected health information was deidentified. The eICU was exempt from institutional review board approval due to its retrospective design, lack of direct patient intervention, and the security scheme, for which the re-identification risk was certified as meeting the safety standards by Privacert (Cambridge, MA) (Health Insurance Portability and Accountability Act Certification no. 1031219-2). Access to the databases can be obtained after completing the mandatory steps as mentioned on the following website, for respectively the MIMIC III and eICU database;

[**https://mimic.physionet.org/gettingstarted/access/**](https://mimic.physionet.org/gettingstarted/access/)**,**

**<https://eicu-crd.mit.edu/gettingstarted/access/>**

**Technical validation**

For the MIMIC-III as stated by Johnson, A.E.W. et al [1]: The number of structural changes were minimized to achieve the desired level of deidentification and data schema, helping to ensure that MIMIC-III closely represents the raw data collected within the Beth Israel Deaconess Medical Center. Best practice for scientific computing was followed where possible. Code used to build MIMIC-III was version controlled and developed collaboratively within the laboratory. This approach encouraged and facilitated sharing of readable code and documentation, as well as frequent feedback from colleagues. Issue tracking is used to ensure that limitations of the data and code are clearly documented and are dealt with as appropriate. The research community is encouraged to report and address issues as they are found, and a system for releasing minor database updates is in place.

For the eICU as stated by Pollard, T.J. et al [4]: Data were verified for integrity during the data transfer process from Philips to MIT using MD5 checksums. In order to maintain data fidelity, very little post-processing has been performed. Each participant hospital in the database has customized workflows and clinical documentation processes, and as a result, the reliability and completion of data elements varies on a hospital and/or ICU level. The data archived within eICU-CRD were intended for use during routine clinical care, and not for secondary analysis. Thus, care must be taken when using the data, as inconsistencies which are inconsequential for clinical care may impact analyses performed. A public issue tracker is used as a forum for reporting technical issues and describing solutions.

**Other data preparation and extraction**

Data was extracted from the database using structured query language (SQL). The data was assessed for completeness and consistency; outliers, defined as an observation that lies outside 1.5 x Interquartile Range (IQR), were checked and substituted by the 5th or 95th percentile.

*Additional description of the mediation analysis*

Mediators are variables that are affected by group assignment and that subsequently can affect the outcome. Therefore, mediators are on the causal pathway of the relation between group and outcome, at least partly explaining the effects of the group on the outcome. For the mediation models, the following estimates are described: 1) the total effect (estimates the total effect of sex on ventilation); 2) the average causal mediation effect (ACME, explains how much of the effect of sex on ventilation is explained by the mediator [height or weight]); 3) the average direct effect (ADE, explains how much of the effect of sex on ventilation is still explained by sex after considering the effect of the mediator); and 4) the proportion of mediation (estimates the proportion of the total effect that is explained by the mediator).

| **Table 1. Baseline Characteristics of the Included Patients** | | | |
| --- | --- | --- | --- |
|  | **Females**  **(*n* = 3614)** | **Males**  **(*n* = 4593)** | ***p* value** |
| Age, years | 65.3 (53.2 – 76.1) | 62.3 (50.0 – 74.0) | < 0.001 |
| ABW, kg | 72.8 (60.0 – 90.3) | 87.0 (74.7 – 103.7) | < 0.001 |
| Body height, cm | 163 (157 – 167) | 178 (172 – 183) | < 0.001 |
| Body mass index, kg/m^2^ | 27.8 (23.3 – 34.6) | 28.3 (24.4 – 33.3) | 0.286 |
| Predicted body weight, kg | 54.7 (50.1 – 58.8) | 73.1 (67.8 – 77.7) | < 0.001 |
| Type of admission |  |  | 0.369 |
| Medical | 3161 (87.5) | 4060 (88.4) |  |
| Surgical elective | 311 (8.6) | 375 (8.2) |  |
| Surgical urgency | 142 (3.9) | 158 (3.4) |  |
| Source of admission |  |  | 0.138 |
| Ward or step–down unit | 647 (17.9) | 772 (16.8) |  |
| Emergency room | 1776 (49.1) | 2341 (51.0) |  |
| Office or operating room | 658 (18.2) | 794 (17.3) |  |
| Transferred from other hospital | 514 (14.2) | 672 (14.6) |  |
| Other | 19 (0.5) | 14 (0.3) |  |
| Initial diagnosis |  |  | < 0.001 |
| Sepsis (including pneumonia) | 973 (28.7) | 1058 (24.7) |  |
| Cardiovascular disease | 536 (15.8) | 820 (19.1) |  |
| Other respiratory condition | 566 (16.7) | 624 (14.6) |  |
| Neurological condition | 660 (19.4) | 927 (21.6) |  |
| Renal condition | 50 (1.5) | 38 (0.9) |  |
| Other | 609 (17.9) | 818 (19.1) |  |
| Chronic obstructive pulmonary disease | 584 (16.2) | 564 (12.3) | < 0.001 |
| Acute respiratory distress syndrome | 389 (10.8) | 481 (10.5) | 0.697 |
| Mild | 49 / 327 (15.0) | 54 / 425 (12.7) |  |
| Moderate | 168 / 327 (51.4) | 225 / 425 (52.9) | 0.832 |
| Severe | 110 / 327 (33.6) | 146 / 425 (34.3) |  |
| Need of vasopressors in the first 24 hours | 1905 (53.1) | 2432 (53.5) | 0.683 |
| Limitation of support at the end of stay | 493 (14.4) | 543 (12.4) | 0.011 |
| Severity of illness |  |  |  |
| Oxford acute severity of illness score | 38 (32 – 43) | 36 (31 – 42) | < 0.001 |
| Sequential organ failure assessment | 6 (4 – 9) | 7 (4 – 9) | < 0.001 |
| Vital signs at beginning of ventilation |  |  |  |
| Heart rate, bpm | 93 (81 – 105) | 92 (80 – 104) | 0.006 |
| Mean arterial pressure, mmHg | 81 (73 – 91) | 81 (73 – 91) | 0.284 |
| SpO_2_, % | 96 (93 – 98) | 96 (94 – 97) | 0.831 |
| Temperature, ºC | 36.9 (36.5 – 37.4) | 37.0 (36.5 – 37.5) | < 0.001 |
| Laboratory data at the beginning of ventilation |  |  |  |
| pH | 7.36 (7.30 – 7.42) | 7.36 (7.30 – 7.41) | 0.153 |
| PaO_2_ / FiO_2_ | 239 (167 – 341) | 230 (160 – 327) | 0.003 |
| PaCO_2_, mmHg | 39.5 (34.5 – 46.2) | 40.5 (36.0 – 46.5) | < 0.001 |
| Data are median (quartile 25% – quartile 75%) or N (%)  *bpm: beats per minute; SpO_2_: pulse oximetry* | | | |

| **Table 2. Baseline Characteristics of the Included Patients According to Tertiles of Year of Admission** | | | | | | |
| --- | --- | --- | --- | --- | --- | --- |
|  | **Females** | | | **Males** | | |
|  | **2001 – 2005**  **(*n* = 749)** | **2006 – 2010**  **(*n* = 701)** | **2011 – 2015**  **(*n* = 2164)** | **2001 – 2005**  **(*n* = 951)** | **2006 – 2010**  **(*n* = 940)** | **2011 – 2015**  **(*n* = 2702)** |
| Age, years | 69.2 (54.6 – 78.9) | 66.2 (51.2 – 77.7) | 64.0 (54.0 – 74.0) | 63.4 (49.2 – 75.9) | 61.8 (49.8 – 73.7) | 62.0 (50.0 – 73.0) |
| ABW, kg | 70.0 (58.0 – 84.0) | 73.0 (60.0 – 89.1) | 74.4 (60.2 – 93.4) | 83.0 (71.7 – 98.2) | 87.0 (75.0 – 103.6) | 88.3 (75.0 – 105.2) |
| Body weight, cm | 163 (157 – 167) | 163 (157 – 165) | 163 (157 – 167) | 178 (170 – 183) | 178 (173 – 183) | 178 (172 – 183) |
| Body mass index, kg/m^2^ | 26.8 (22.4 – 31.6) | 28.4 (23.9 – 33.5) | 28.0 (23.3 – 35.5) | 27.4 (24.2 – 31.7) | 28.6 (24.8 – 33.5) | 28.4 (24.3 – 33.5) |
| Predicted body weight, kg | 54.7 (50.1 – 59.4) | 54.7 (50.1 – 57.1) | 54.7 (50.1 – 58.9) | 73.1 (66.2 – 77.7) | 73.1 (68.5 – 77.7) | 73.1 (67.8 – 77.7) |
| Type of admission |  |  |  |  |  |  |
| Medical | 629 (84.0) | 627 (89.4) | 1905 (88.0) | 824 (86.6) | 856 (91.1) | 2380 (88.1) |
| Surgical elective | 52 (6.9) | 60 (8.6) | 199 (9.2) | 83 (8.7) | 60 (6.4) | 232 (8.6) |
| Surgical urgency | 68 (9.1) | 14 (2.0) | 60 (2.8) | 44 (4.6) | 24 (2.6) | 90 (3.3) |
| Source of admission |  |  |  |  |  |  |
| Ward or step–down unit | 25 (3.3) | 68 (9.7) | 554 (25.6) | 22 (2.3) | 94 (10.0) | 656 (24.3) |
| Emergency room | 410 (54.7) | 384 (54.8) | 982 (45.4) | 576 (60.6) | 518 (55.1) | 1247 (46.2) |
| Office or operating room | 88 (11.7) | 74 (10.6) | 496 (22.9) | 112 (11.8) | 83 (8.8) | 599 (22.2) |
| Transferred from other hospital | 212 (28.3) | 175 (25.0) | 127 (5.9) | 232 (24.4) | 244 (26.0) | 196 (7.3) |
| Other | 14 (1.9) | 0 (0.0) | 5 (0.2) | 9 (0.9) | 1 (0.1) | 4 (0.1) |
| Initial diagnosis |  |  |  |  |  |  |
| Sepsis (including pneumonia) | 155 (20.7) | 168 (24.0) | 650 (33.4) | 144 (15.1) | 209 (22.2) | 705 (29.4) |
| Cardiovascular disease | 173 (23.1) | 130 (18.5) | 233 (12.0) | 240 (25.2) | 232 (24.7) | 348 (14.5) |
| Other respiratory condition | 115 (15.4) | 128 (18.3) | 323 (16.6) | 130 (13.7) | 134 (14.3) | 360 (15.0) |
| Neurological condition | 127 (17.0) | 121 (17.3) | 412 (21.2) | 196 (20.6) | 175 (18.6) | 556 (23.2) |
| Renal condition | 12 (1.6) | 8 (1.1) | 30 (1.5) | 12 (1.3) | 5 (0.5) | 21 (0.9) |
| Other | 167 (22.3) | 146 (20.8) | 296 (15.2) | 229 (24.1) | 185 (19.7) | 404 (16.9) |
| Chronic obstructive pulmonary disease | 49 (6.5) | 39 (5.6) | 496 (22.9) | 50 (5.3) | 45 (4.8) | 469 (17.4) |
| Acute respiratory distress syndrome | 87 (11.6) | 68 (9.7) | 234 (10.8) | 127 (13.4) | 95 (10.1) | 259 (9.6) |
| Mild | 10 (1.3) | 9 (1.3) | 30 (1.4) | 8 (0.8) | 10 (1.1) | 36 (1.4) |
| Moderate | 44 (5.9) | 33 (4.7) | 91 (4.3) | 65 (6.8) | 57 (6.1) | 103 (3.9) |
| Severe | 33 (4.4) | 26 (3.7) | 51 (2.4) | 54 (5.7) | 28 (3.0) | 64 (2.4) |
| Need of vasopressors in the first 24 hours | 357 (47.7) | 349 (49.8) | 1199 (56.0) | 441 (46.4) | 506 (53.8) | 1485 (56.0) |
| Limitation of support at the end of stay | 231 (30.9) | 156 (24.8) | 106 (5.2) | 250 (26.5) | 187 (21.4) | 106 (4.1) |
| Severity of illness |  |  |  |  |  |  |
| Oxford acute severity of illness score | 39 (34 – 44) | 39 (34 – 44) | 37 (31 – 42) | 37 (31 – 42) | 38 (32 – 44) | 36 (30 – 42) |
| Sequential organ failure assessment | 5 (4 – 8) | 6 (3 – 9) | 6 (4 – 9) | 6 (4 – 8) | 7 (4 – 10) | 7 (5 – 10) |
| Vital signs at beginning of ventilation |  |  |  |  |  |  |
| Heart rate, bpm | 94 (82 – 105) | 92 (80 – 106) | 92 (81 – 105) | 93 (81 – 103) | 91 (80 – 104) | 91 (80 – 104) |
| Mean arterial pressure, mmHg | 82 (73 – 91) | 78 (72 – 88) | 81 (73 – 92) | 82 (74 – 91) | 79 (73 – 87) | 82 (74 – 92) |
| SpO_2_, % | 96 (93 – 98) | 97 (95 – 98) | 95 (93 – 97) | 96 (94 – 98) | 96 (94 – 98) | 95 (93 – 97) |
| Temperature, ºC | 37.1 (36.6 – 37.6) | 37.0 (36.5 – 37.5) | 36.9 (36.4 – 37.3) | 37.2 (36.7 – 37.7) | 37.1 (36.5 – 37.6) | 37.0 (36.5 – 37.5) |
| Laboratory data at the beginning of ventilation |  |  |  |  |  |  |
| pH | 7.37 (7.33 – 7.42) | 7.37 (7.32 – 7.42) | 7.35 (7.29 – 7.42) | 7.38 (7.33 – 7.42) | 7.36 (7.30 – 7.41) | 7.35 (7.29 – 7.40) |
| PaO_2_ / FiO_2_ | 290 (197 – 399) | 260 (185 – 364) | 212 (148 – 307) | 267 (192 – 373) | 249 (178 – 339) | 210 (144 – 305) |
| PaCO_2_, mmHg | 38.5 (33.7 – 43.5) | 40.0 (35.5 – 45.0) | 40.0 (34.5 – 48.0) | 39.5 (35.1 – 44.5) | 40.5 (36.5 – 45.5) | 41.0 (36.0 – 48.0) |
| Data are median (quartile 25% – quartile 75%) or N (%)  *bpm: beats per minute; SpO_2_: pulse oximetry* | | | | | | |

| **Table 3. Ventilatory Parameters in the First Two days of Ventilation According to Tertiles of Year of Admission*** | | | | | | | | |
| --- | --- | --- | --- | --- | --- | --- | --- | --- |
|  | **Females** | | | **Males** | | |  |  |
|  | **2001 – 2005**  **(*n* = 749)** | **2006 – 2010**  **(*n* = 701)** | **2011 – 2015**  **(*n* = 2164)** | **2001 – 2005**  **(*n* = 951)** | **2006 – 2010**  **(*n* = 940)** | **2011 – 2015**  **(*n* = 2702)** | ***p* value**  **(Sex)**** | ***p* value**  **(Trend)**** |
| First day of ventilation |  |  |  |  |  |  |  |  |
| Tidal volume |  |  |  |  |  |  |  |  |
| Absolute, mL | 535 (500 – 600) | 500 (450 – 525) | 450 (400 – 500) | 647 (600 – 705) | 566 (514 – 615) | 516 (493 – 574) | < 0.001 | < 0.001 |
| Mode | 500 | 500 | 500 | 600 | 600 | 500 | --- | --- |
| mL/kg PBW | 10.2 (9.1 – 11.4) | 9.0 (8.2 – 10.0) | 8.2 (7.5 – 9.1) | 9.2 (8.2 – 10.1) | 8.0 (7.3 – 8.8) | 7.3 (6.6 – 8.0) | < 0.001 | 0.069 |
| Mode | 13.2 | 9.1 | 8.0 | 11.3 | 7.5 | 6.8 | --- | --- |
| mL/kg ABW | 7.8 (6.5 – 9.0) | 6.6 (5.5 – 7.9) | 6.0 (4.9 – 7.3) | 7.7 (6.5 – 8.8) | 6.5 (5.6 – 7.6) | 5.9 (5.0 – 7.0) | 0.636 | 0.301 |
| Mode | 8.0 | 9.0 | 5.9 | 6.1 | 6.2 | 5.5 | --- | --- |
| PEEP, cmH_2_O | 5 (5 – 8) | 5 (5 – 9) | 5 (5 – 8) | 5 (5 – 8) | 5 (5 – 9) | 5 (5 – 8) | 0.171 | 0.715 |
| Respiratory rate, mpm | 18 (15 – 21) | 19 (17 – 22) | 16 (14 – 20) | 18 (16 – 21) | 20 (17 – 23) | 16 (14 – 20) | 0.084 | 0.079 |
| Plateau pressure, cmH_2_O | 22 (18 – 26) | 21 (18 – 25) | 20 (17 – 25) | 21 (18 – 25) | 20 (16 – 24) | 19 (16 – 23) | 0.637 | 0.021 |
| Driving pressure, cmH_2_O | 15 (13 – 19) | 14 (12 – 17) | 14 (11 – 18) | 14 (12 – 17) | 13 (10 – 16) | 13 (10 – 16) | 0.102 | 0.031 |
| Peak pressure, cmH_2_O | 28 (23 – 33) | 25 (21 – 30) | 25 (20 – 30) | 28 (23 – 32) | 25 (21 – 30) | 23 (19 – 28) | 0.034 | < 0.001 |
| Minute ventilation, L/min | 9.6 (8.4 – 11.3) | 9.3 (8.2 – 10.7) | 7.6 (6.3 – 9.2) | 11.6 (10.1 – 13.3) | 11.2 (9.8 – 13.0) | 8.9 (7.5 – 10.7) | < 0.001 | < 0.001 |
| L/min PBW | 0.18 (0.16 – 0.21) | 0.17 (0.15 – 0.20) | 0.14 (0.12 – 0.17) | 0.16 (0.14 – 0.19) | 0.16 (0.14 – 0.18) | 0.12 (0.10 – 0.15) | < 0.001 | 0.950 |
| Mechanical power, J/min | 18.3 (14.6 – 22.9) | 16.4 (13.0 – 20.7) | 12.8 (9.3 – 17.3) | 22.2 (17.5 – 28.5) | 19.5 (15.0 – 25.2) | 14.0 (10.4 – 19.2) | < 0.001 | < 0.001 |
| J/min PBW | 0.35 (0.28 – 0.44) | 0.31 (0.24 – 0.39) | 0.24 (0.17 – 0.32) | 0.33 (0.26 – 0.41) | 0.28 (0.21 – 0.38) | 0.20 (0.14 – 0.27) | 0.040 | 0.073 |
| Ventilatory ratio | 2.12 (1.75 - 2.57) | 2.09 (1.69 - 2.590 | 1.80 (1.39 - 2.34) | 1.98 (1.65 - 2.40) | 1.89 (1.59 - 2.30) | 1.63 (1.30 - 2.07) | 0.003 | 0.339 |
| Second day of ventilation |  |  |  |  |  |  |  |  |
| Tidal volume |  |  |  |  |  |  |  |  |
| Absolute, mL | 512 (466 – 600) | 475 (425 – 516) | 450 (400 – 500) | 618 (555 – 700) | 556 (502 – 614) | 507 (483 – 565) | < 0.001 | 0.020 |
| Mode | 500 | 500 | 500 | 600 | 600 | 500 | --- | --- |
| mL/kg PBW | 9.9 (8.8 – 11.0) | 8.8 (8.0 – 9.9) | 8.0 (7.3 – 9.1) | 9.0 (7.9 – 9.9) | 7.8 (7.1 – 8.7) | 7.3 (6.6 – 7.9) | < 0.001 | 0.052 |
| Mode | 13.2 | 8.2 | 7.9 | 9.0 | 7.5 | 6.8 | --- | --- |
| mL/kg ABW | 7.6 (6.2 – 8.8) | 6.4 (5.3 – 7.8) | 5.9 (4.8 – 7.2) | 7.4 (6.3 – 8.6) | 6.4 (5.4 – 7.5) | 5.9 (4.9 – 6.9) | 0.957 | 0.740 |
| Mode | 10.0 | 8.0 | 5.9 | 6.1 | 6.7 | 5.5 | --- | --- |
| PEEP, cmH_2_O | 5 (5 – 8) | 5 (5 – 10) | 5 (5 – 8) | 5 (5 – 10) | 6 (5 – 10) | 5 (5 – 8) | 0.004 | 0.119 |
| Respiratory rate, mpm | 18 (15 – 21) | 19 (16 – 22) | 16 (14 – 21) | 18 (15 – 22) | 20 (17 – 23) | 16 (14 – 20) | 0.040 | 0.047 |
| Plateau pressure, cmH_2_O | 22 (18 – 26) | 21 (17 – 24) | 20 (17 – 24) | 21 (18 – 25) | 20 (17 – 24) | 19 (16 – 23) | 0.485 | 0.006 |
| Driving pressure, cmH_2_O | 15 (12 – 18) | 13 (11 – 16) | 13 (11 – 17) | 14 (11 – 17) | 13 (10 – 15) | 12 (10 – 15) | 0.122 | 0.071 |
| Peak pressure, cmH_2_O | 27 (23 – 32) | 25 (21 – 30) | 24 (20 – 29) | 28 (23 – 32) | 25 (21 – 30) | 23 (19 – 28) | 0.003 | < 0.001 |
| Minute ventilation, L/min | 9.5 (8.0 – 10.9) | 9.1 (7.9 – 10.3) | 7.5 (6.2 – 9.2) | 11.2 (9.2 – 13.2) | 11.0 (9.5 – 12.7) | 8.8 (7.2 – 10.8) | < 0.001 | < 0.001 |
| L/min PBW | 0.18 (0.15 – 0.21) | 0.17 (0.15 – 0.20) | 0.14 (0.11 – 0.17) | 0.16 (0.14 – 0.19) | 0.15 (0.13 – 0.18) | 0.12 (0.10 – 0.15) | < 0.001 | 0.945 |
| Mechanical power, J/min | 18.0 (13.9 – 22.7) | 16.3 (12.3 – 20.7) | 12.3 (9.2 – 17.0) | 22.0 (17.0 – 28.8) | 19.9 (15.0 – 25.7) | 14.0 (10.3 – 19.2) | < 0.001 | < 0.001 |
| J/min PBW | 0.34 (0.27 – 0.43) | 0.30 (0.24 – 0.39) | 0.23 (0.17 – 0.31) | 0.33 (0.25 – 0.42) | 0.28 (0.21 – 0.37) | 0.19 (0.14 – 0.27) | 0.652 | 0.008 |
| Ventilatory ratio | 1.86 (1.58 - 2.28) | 1.87 (1.54 - 2.27) | 1.67 (1.31 - 2.14) | 1.77 (1.47 - 2.14) | 1.74 (1.42 - 2.05) | 1.48 (1.19 - 1.88) | 0.072 | 0.353 |
| Data are median (quartile 25% – quartile 75%)  *ABW: actual body weight; mpm: movements per minute; PBW: predicted body weight; PEEP: positive end–expiratory pressure*  * Data was aggregated as the median of 4 values per day (measured every 6 hours)  ** *p* value from a mixed–effect linear model with dataset, sex and interaction between sex and year tertiles as fixed effect and hospitals as random effect. *p* values (sex) reflect the overall test for difference between sex across the years while *p* values (trend) evaluate if change over time differed by sex | | | | | | | | |

| **Table 4. Effect on Low Tidal Volume Ventilation According to Tertiles of Year of Admission** | | | | | | |
| --- | --- | --- | --- | --- | --- | --- |
|  | **2001 – 2005** | | **2006 – 2010** | | **2011 – 2015** | |
|  | **Absolute Difference**  **(95% CI)** | ***p* value** | **Absolute Difference**  **(95% CI)** | ***p* value** | **Absolute Difference**  **(95% CI)** | ***p* value** |
| Female sex | 1.02 (-4.80 to 6.85) | 0.730 | 5.11 (-1.97 to 12.19) | 0.157 | -2.10 (-5.55 to 1.35) | 0.233 |
| Age | -2.13 (-4.35 to 0.09) | 0.060 | -3.62 (-6.37 to -0.86) | 0.010 | -0.09 (-1.42 to 1.23) | 0.891 |
| ABW | -9.99 (-12.32 to -7.67) | < 0.001 | -8.96 (-11.77 to -6.15) | < 0.001 | -7.52 (-8.94 to -6.10) | < 0.001 |
| Body height | 15.33 (12.31 to 18.35) | < 0.001 | 28.53 (24.77 to 32.30) | < 0.001 | 23.42 (21.59 to 25.26) | < 0.001 |
| OASIS | 0.51 (-2.05 to 3.08) | 0.695 | 3.95 (0.77 to 7.12) | 0.015 | 1.32 (-0.21 to 2.86) | 0.091 |
| SOFA | 2.84 (0.31 to 5.38) | 0.028 | -0.45 (-3.69 to 2.78) | 0.784 | -1.78 (-3.29 to -0.27) | 0.021 |
| Type of admission |  |  |  |  |  |  |
| Elective surgery | 0 (Reference) | --- | 0 (Reference) | --- | 0 (Reference) | --- |
| Medical admission | 4.27 (-2.87 to 11.41) | 0.241 | 5.22 (-3.93 to 14.38) | 0.263 | 10.12 (5.68 to 14.58) | < 0.001 |
| Urgent surgery | -0.07 (-10.56 to 10.42) | 0.989 | 2.22 (-15.37 to 19.80) | 0.805 | 5.78 (-2.33 to 13.91) | 0.163 |
| pH | -1.84 (-4.22 to 0.55) | 0.131 | 0.79 (-2.20 to 3.79) | 0.603 | 0.14 (-1.33 to 1.61) | 0.848 |
| PaCO_2_ | 7.22 (4.87 to 9.56) | < 0.001 | 9.41 (6.64 to 12.18) | < 0.001 | 4.68 (3.20 to 6.16) | < 0.001 |
| Use of titrated tidal volume | -8.30 (-12.61 to -3.98) | < 0.001 | 0.63 (-4.42 to 5.68) | 0.806 | -3.08 (-5.78 to -0.37) | 0.026 |
| *OASIS: Oxford acute severity of illness score; SOFA: Sequential Organ Failure Assessment* | | | | | | |

**Fig 1. Study flowchart**

**
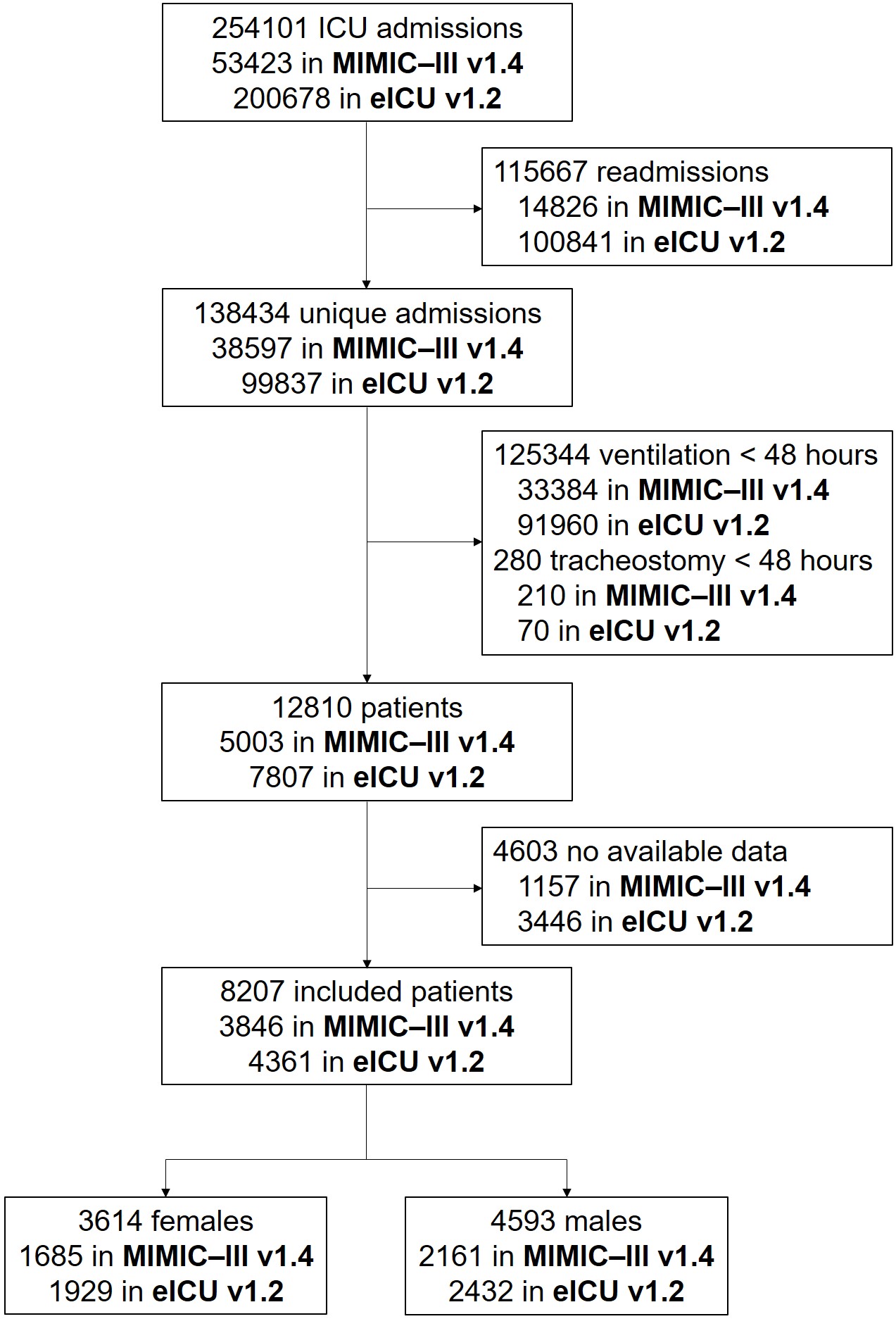
**

**Fig 2. The trend of height and weight during the years**

**
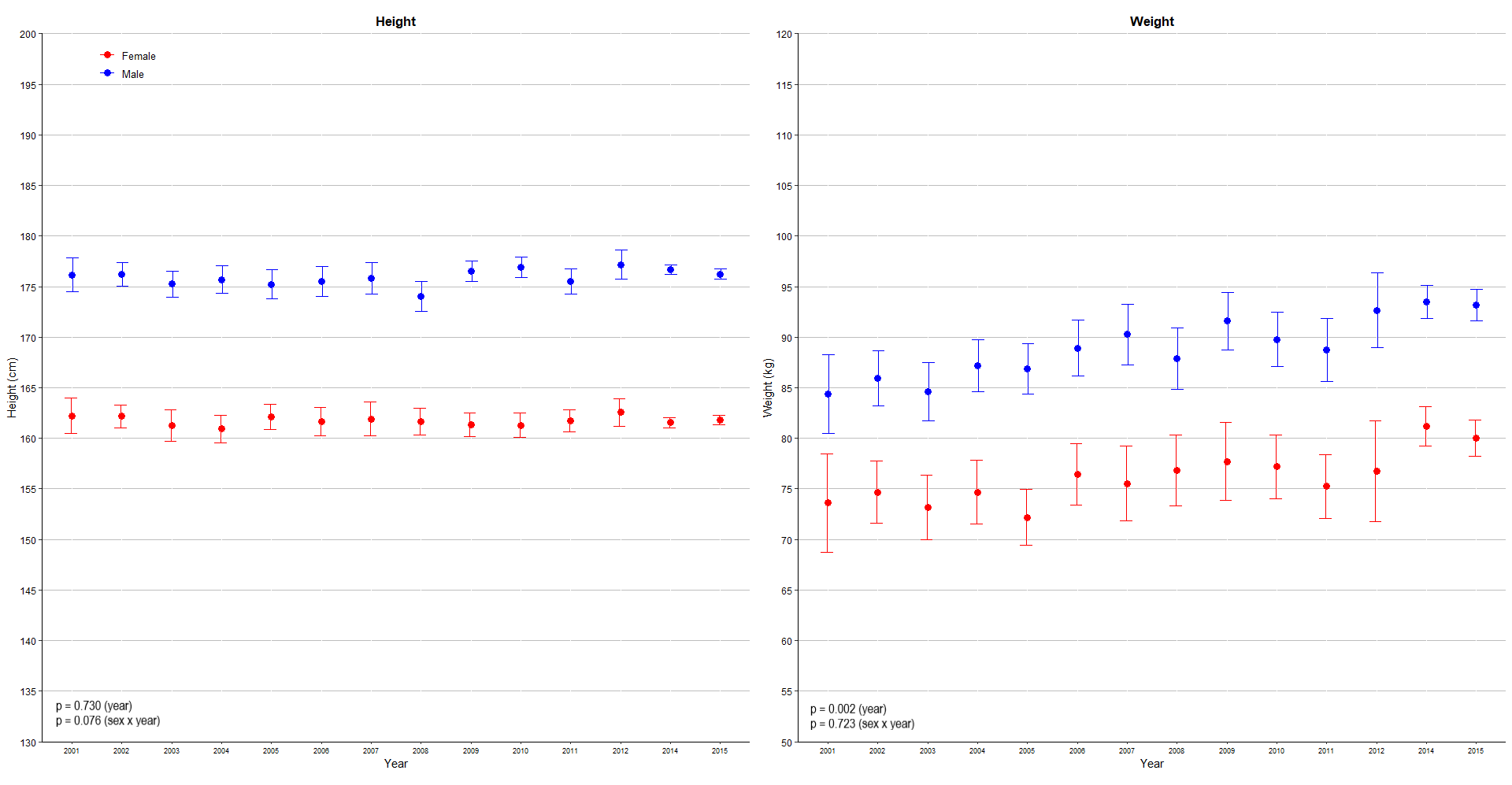
**

*p* values for the year reflect the overall test for difference between years while *p* values for the sex x year interaction evaluate if change over time differed by sex. There is no interaction between dataset and year (*p* = 0.152, and *p* = 0.703 for height and weight, respectively), or dataset and sex x year interaction (*p* = 0.152, and *p* = 0.704 for height and weight, respectively).

**Fig 3. Percentage of patients receiving default tidal volume**


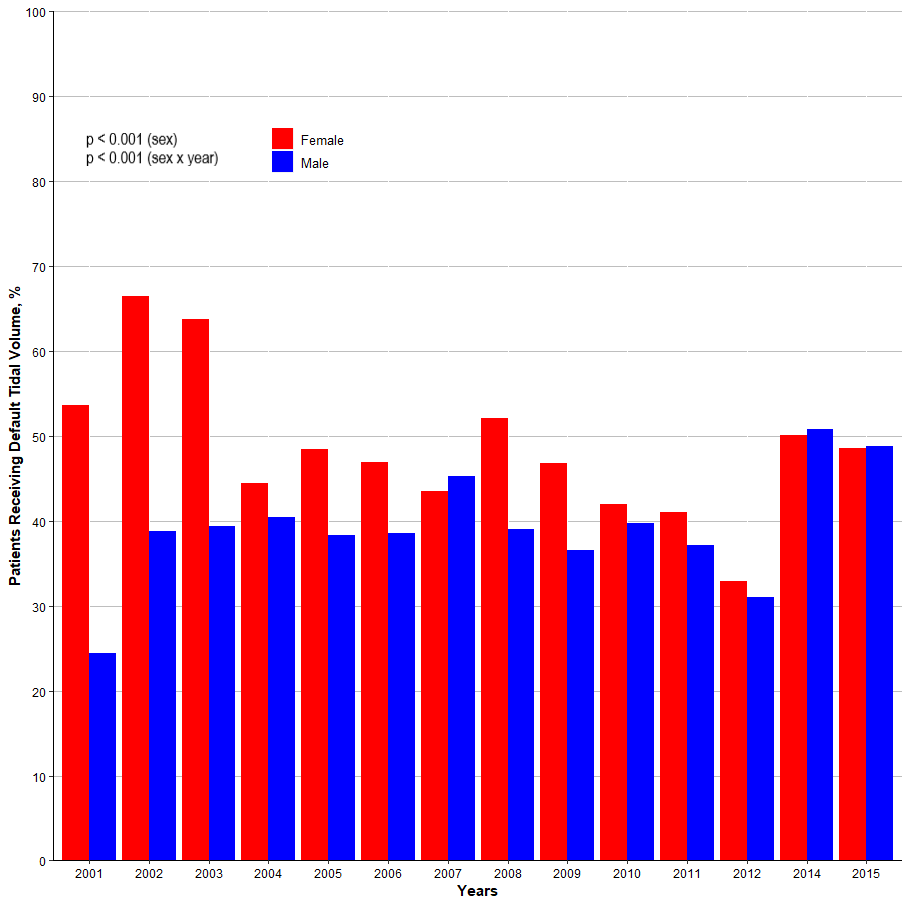


Default tidal volume defined as if the first two reported absolute V_t_  were ‘rounded numbers’. i.e., a V_t_ of 200, 250, 300, 350, 400, 450, 500, 550, 600 or 650 ml. *p* values for sex reflect the overall test for difference between sex over the years while *p* values for the sex x year interaction evaluate if change over time differed by sex

**Fig 4. Absolute tidal volume and corrected tidal volume (by predicted body weight or absolute body weight) over years and according to tertiles of year in patients with sepsis**


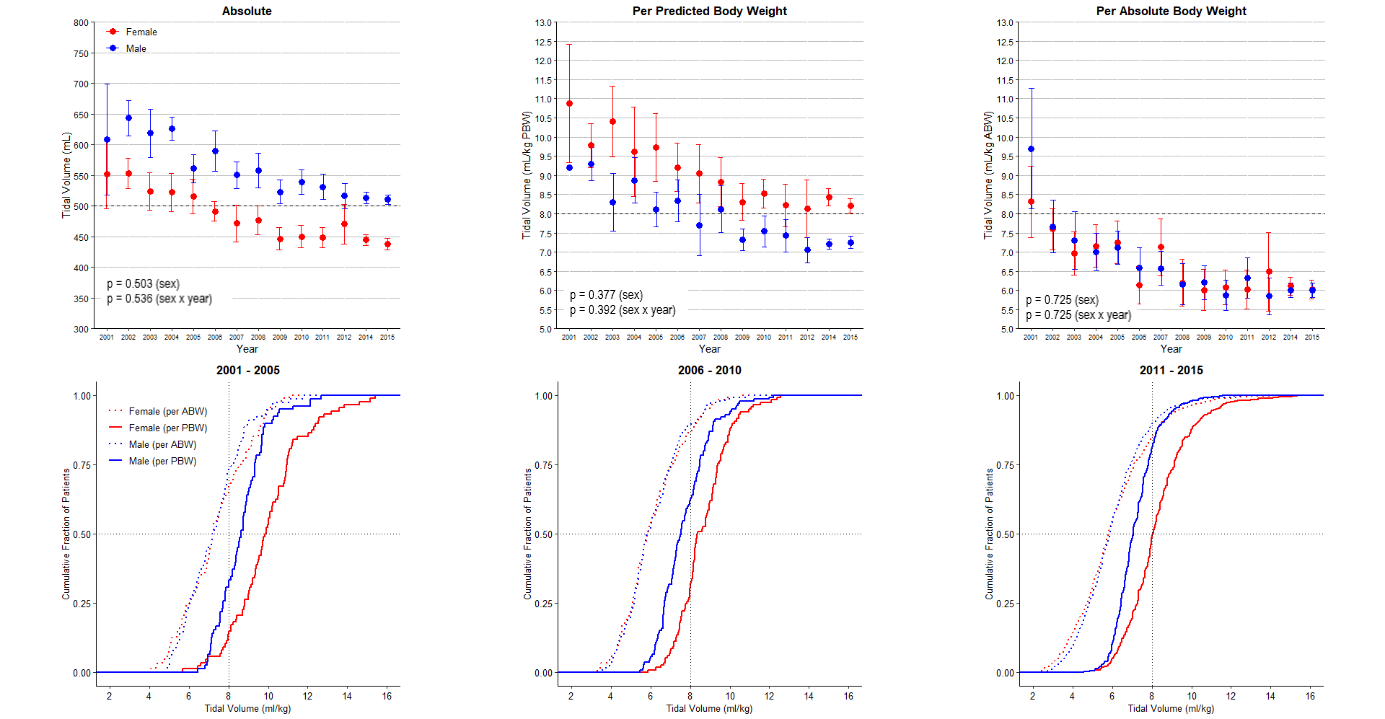


*PBW: predicted body weight; ABW: actual body weight* *p* values for the sex reflect the overall test for difference between sex over the years while *p* values for the sex x year interaction evaluate if change over time differed by sex.

**Fig 5. Absolute tidal volume and corrected tidal volume (by predicted body weight or absolute body weight) over years during controlled vs assisted ventilation in the MIMIC-III database**


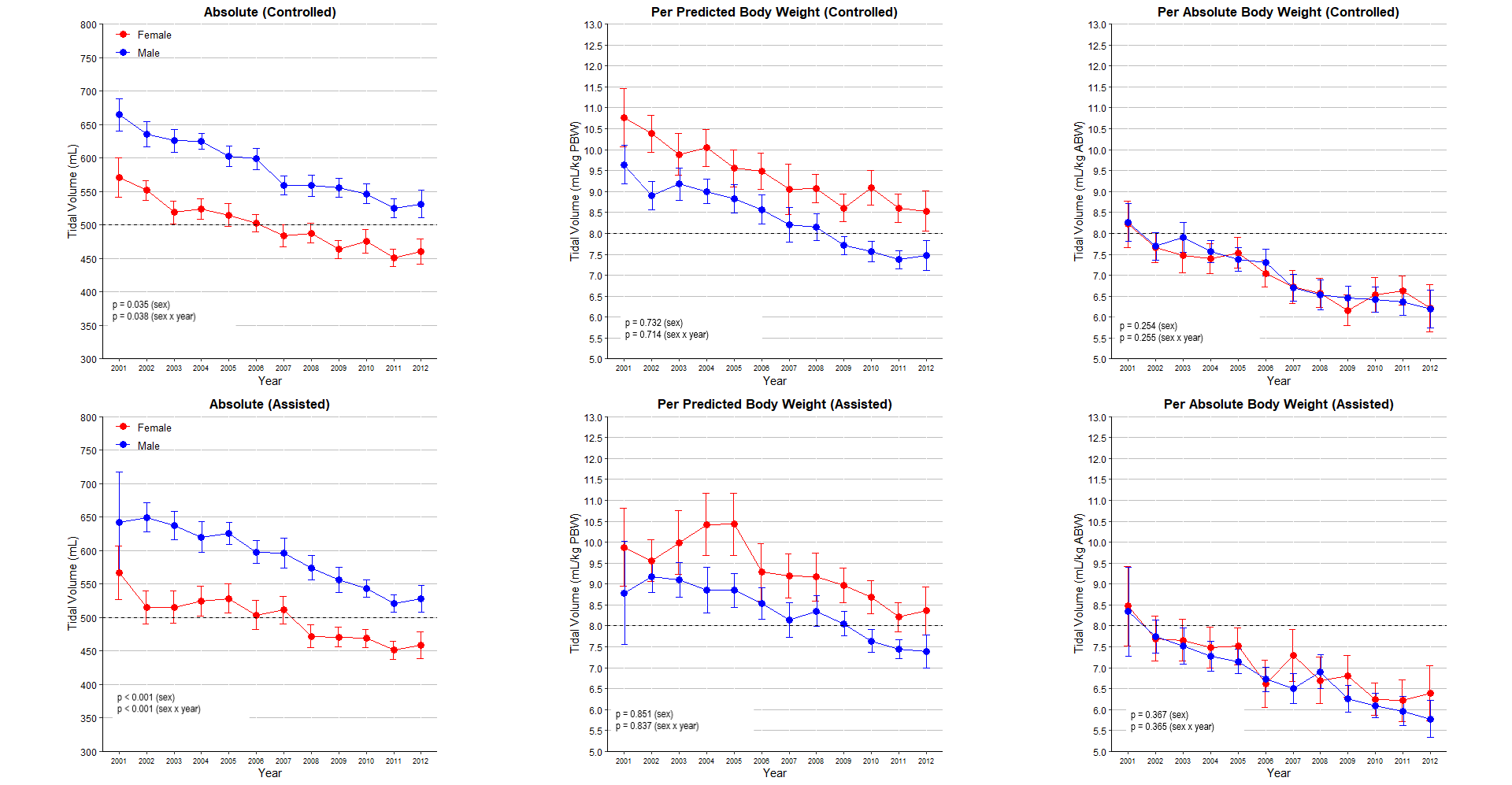


*PBW: predicted body weight; ABW: actual body weight* *p* values for the sex reflect the overall test for difference between sex over the years while *p* values for the sex x year interaction evaluate if change over time differed by sex.

**References**

1. Johnson AE, Pollard TJ, Shen L, Lehman LW, Feng M, Ghassemi M, et al. MIMIC-III, a freely accessible critical care database. Sci Data. 2016;3:160035.

2. Johnson AE, Stone DJ, Celi LA, Pollard TJ. The MIMIC Code Repository: enabling reproducibility in critical care research. J Am Med Inform Assoc. 2018;25(1):32-9.

3. Goldberger AL, Amaral LA, Glass L, Hausdorff JM, Ivanov PC, Mark RG, et al. PhysioBank, PhysioToolkit, and PhysioNet: components of a new research resource for complex physiologic signals. Circulation. 2000;101(23):E215-20.

4. Pollard TJ, Johnson AEW, Raffa JD, Celi LA, Mark RG, Badawi O. The eICU Collaborative Research Database, a freely available multi-center database for critical care research. Scientific Data. 2018;5(1):180178.
