# Supplementary material for: Impact of sex on use of low tidal volume ventilation in invasively ventilated ICU patients—A mediation analysis using two observational cohorts
Source: PLoS One. 2021 Jul 14;16(7):e0253933. doi: 10.1371/journal.pone.0253933 (PMC8279424; doi:10.1371/journal.pone.0253933)
Supplement: Attachment — Submitted filename: PONE-D-21-08610.docx [file pone.0253933.s002.docx]

**Editor**

1. Please ensure that your manuscript meets PLOS ONE’s style requirements, including those for file naming.

**Thank you for your advice. We adjusted the manuscript so that it meets the style requirements.**

2. Please provide additional details regarding participant consent. For each patient cohort, in the ethics statement in the Methods and online submission information, please ensure that you have specified (1) whether consent was informed and (2) what type you obtained (for instance, written or verbal, and if verbal, how it was documented and witnessed). If your study included minors, state whether you obtained consent from parents or guardians. If the need for consent was waived by the ethics committee, please include this information.

**Thank you. We adjusted the paragraph in the Methods dealing with this matter, as follows:**

*‘The Institutional Review Board of the Beth Israel Deaconess Medical Center (2001–P–001699/14) and the Massachusetts Institute of Technology (No. 0403000206) approved the MIMIC project. Requirement for individual patient consent was waived because the project did not impact clinical care and all protected health information was deidentified. The eICU was exempt from institutional review board approval due to its retrospective design, lack of direct patient intervention, and the security scheme, for which the re–identification risk was certified as meeting the safety standards by Privacert (Cambridge, MA) (Health Insurance Portability and Accountability Act Certification no. 1031219-2).’* (Page 7; line 161)

3. We note that you have provided funding information that is not currently declared in your Funding Statement. However, funding information should not appear in the Acknowledgments section or other areas of your manuscript. We will only publish funding information present in the Funding Statement section of the online submission form. Please remove any funding-related text from the manuscript and let us know how you would like to update your Funding Statement. Currently, your Funding Statement reads as follows: ‘The author(s) received no specific funding for this work.’ Please include your amended statements within your cover letter; we will change the online submission form on your behalf.

**In line with your remarks we added the following funding statement:**

‘*The authors received no specific funding for this work.’*

4. Ary Serpa Neto reported receiving personal fees from Dräger outside of the submitted work. Marcelo Gama de Abreu reported receiving grants and personal fees from Drägerwerk AG and GlaxoSmithKline and receiving personal fees from GE Healthcare outside of the submitted work. No other disclosures were reported. We note that you received funding from a commercial source: Drägerwerk A G, GlaxoSmithKline, GE Healthcare. Please provide an amended Competing Interests Statement that explicitly states this commercial funder, along with any other relevant declarations relating to employment, consultancy, patents, products in development, marketed products, etc. Within this Competing Interests Statement, please confirm that this does not alter your adherence to all PLOS ONE policies on sharing data and materials by including the following statement: ‘This does not alter our adherence to PLOS ONE policies on sharing data and materials.’ (as detailed online in our guide for authors http://journals.plos.org/plosone/s/competing-interests). If there are restrictions on sharing of data and/or materials, please state these. Please note that we cannot proceed with consideration of your article until this information has been declared.

**We confirm that this does not alter the adherence to PLOS ONE policies on sharing data and materials by including the statement you referred to. We also declare that we received no funding related to the submitted work and I therefore I added the following statement to the Conflict of interest paragraph.**

*‘This does not alter our adherence to PLOS ONE policies on sharing data and materials.’* (Page 23; line 473)

5. We noted in your submission details that a portion of your manuscript may have been presented or published elsewhere. ‘Gender Differences in Tidal Volume Limitation – an ICU–database analysis. Oral presentation at European Respiratory Society Annual International congress 2020, Online’. Please clarify whether this conference proceeding or publication was peer–reviewed and formally published. If this work was previously peer–reviewed and published, in the cover letter please provide the reason that this work does not constitute dual publication and should be included in the current manuscript.

**Indeed, we reported part of the results of a preliminary analysis of the two databases at the congress mentioned. This was neither peer–reviewed, nor formally published and therefore does not constitute dual publication.**

6. We note that you have indicated that data from this study are available upon request. PLOS only allows data to be available upon request if there are legal or ethical restrictions on sharing data publicly. For information on unacceptable data access restrictions, please see <http://journals.plos.org/plosone/s/data-availability#loc-unacceptable-data-access-restrictions>. In your revised cover letter, please address the following prompts: 6a) If there are ethical or legal restrictions on sharing a de-identified data set, please explain them in detail (e.g., data contain potentially identifying or sensitive patient information) and who has imposed them (e.g., an ethics committee). Please also provide contact information for a data access committee, ethics committee, or other institutional body to which data requests may be sent. 6b) If there are no restrictions, please upload the minimal anonymized data set necessary to replicate your study findings as either Supporting Information files or to a stable, public repository and provide us with the relevant URLs, DOIs, or accession numbers. Please see <http://www.bmj.com/content/340/bmj.c181.long> for guidelines on how to de-identify and prepare clinical data for publication. For a list of acceptable repositories, please see <http://journals.plos.org/plosone/s/data-availability#loc-recommended-repositories>.

**There are no legal or ethical restrictions on sharing the data publicly. However, some steps must be taken before other external researchers can re-use the data – we ourselves are not the owner the 2 databases. Please advise us on how we should proceed here?**

**Access to the eICU Collaborative Research Database starts with Completing the required CITI training course ‘Data or Specimens Only Research’ course. Follow the steps as mentioned on the website using the following link;** **<https://eicu-crd.mit.edu/gettingstarted/access/> On this website one can also see the steps needed to Request access to the eICU Collaborative Research Database after completing the course. It starts with registering for an account on PhysioNet.**

**For the MIMIC III database it is more or less the same. Researchers seeking to use the database must formally request access with the steps mentioned on the website using the following link;** **<https://mimic.physionet.org/gettingstarted/access/> The same training course as for the eICU database must be completed and also on this website you can see the steps that must be taken to request access.**

**We added the information above to the online supplement, as follows:**

*‘Access to the databases can be obtained after completing the mandatory steps as mentioned on the following website, for respectively the MIMIC III and eICU database:*

[*https://mimic.physionet.org/gettingstarted/access/*](https://mimic.physionet.org/gettingstarted/access/) *and* [*https://eicu-crd.mit.edu/gettingstarted/access/*](https://eicu-crd.mit.edu/gettingstarted/access/)*.’* (Supplement Page 4; line 63)

7. Please include captions for your Supporting Information files at the end of your manuscript, and update any in-text citations to match accordingly. Please see our Supporting Information guidelines for more information: <http://journals.plos.org/plosone/s/supporting-information>.

**This is now included.**

8. Please review your reference list to ensure that it is complete and correct. If you have cited papers that have been retracted, please include the rationale for doing so in the manuscript text, or remove these references and replace them with relevant current references. Any changes to the reference list should be mentioned in the rebuttal letter that accompanies your revised manuscript. If you need to cite a retracted article, indicate the article’s retracted status in the References list and also include a citation and full reference for the retraction notice.

**The reference list is now complete and correct.**

**Reviewer 1**

1. This is a pothoc analysis of two large longitudinal projects from 59 ICUs in USA including 8207 patients with mechanical ventilation > 48 hours. Temporal changes of Vt and gender differences were analyzed during the period 2001-2015 . The authors did not included more recent data (2015-2020) with probably higher implementació of protective ventilation Vt declines over the years in both sex but with a persistent difference between females (Vt 10.2 to 8.2) vs males (Vt 9.2 to 7.3) ml/kg PBW.

What is the real clinical impact and relevance of a gender difference of median Vt 0.9 ml/KgIBW among both groups ? What is the Vt atributable mortality considering that the majority of respiratory mechanical parameters were in the recommended level of protective mechanical ventilation and no gender differences were observed except for minute ventilation? What was ventilatory ratio in both groups?

What is the Vt atributable mortality considering that the majority of respiratory mechanical parameters were in the recommended level of protective mechanical ventilation and no gender differences were observed except for minute ventilation?

**Thank you for these questions. First, we would like to stress that we are reporting the data in medians (and interquartile ranges), and comparing only medians may give a false impression that the temporal changes are not so ‘impressive’. Indeed, one should also compare e.g., the proportions of patients that did, or did not receive ‘low tidal volume ventilation’––as you can see, the difference in proportions of patients that did receive LTVV changed, and also that differences persisted.**

**We agree it would be interesting to understand the ‘real’ clinical impact and relevance of the (temporal change in) gender difference––this, however, was not the aim of this study. It is even impossible to study this with the 2 cohorts used by us, as there are certainly temporal changes in other care processes that (also) impact outcome.**

**We added the following to the Discussion:**

*‘**There is abundant and convincing evidence that the use of an incorrect (i.e., too high) V_T_ is associated with higher mortality and morbidity in ICU patients [21, 29, 30, 42-44]. In fact, V_T_ is one of the key elements of lung–protective ventilation. Critically ill females tend to develop ARDS more often, and also have higher mortality rates [21, 29]. In addition, in female patients after cardiac surgery, a higher V_T_ was found to have independent associations with organ dysfunction and prolonged ICU stay [30]. Whether the temporal changes in the current analysis translate in better outcomes, and whether this is different for females and males, cannot be reliably determined since we could not correct for temporal changes in other care processes that certainly have an impact on outcome as well.’* (Page 20; line 396)

**Concerning your comment regarding the ventilatory ratio; you are right, there was also a (in part understandable) difference in minute volume – interestingly, there was a difference in ventilation ratio, we added this to table 1** (Page 13) **and S3 table** (Supplement Page 10).

2. How and when actual body weigth and heigth were messured?

**Thank you for this question. These data were either data from before the ICU admission as recorded in the medical records, and if unknown data collected at the day of ICU admission. This is now added to the Methods, as follows:**

*‘**Body weight and height were collected from the medical records, i.e., the body weight and height as used for decisions in care. These could either be based on measurement at earlier contacts with a patient, measurements in the ward, or at admission to the ICU.’* (Page 8; line 182)

3. The authors used a hight resolution data base. Could you please explain what you mean by high resolution? What is the quality control of the data base?

**We agree ‘high resolution’ could mean many things, and therefore replaced ‘high resolution’ with ‘temporal’, as follows:**

‘*An elaborative description of these temporal databases can be found in the S1 additional Methods’* (Page 7; line 157)

*‘Strengths of this analysis are the use of 2 conventionally–sized temporal databases that contain granular ventilation data of two well–defined cohorts of patients from several centres from 2001 to 2015.’* (Page 18; line 349)

**Concerning your comment regarding the quality control of the data bases, for both the MIMIC-III and the eICU a paper has been published in addressing the technical validation, this is now added to the supplement, as follows:**

*‘For the MIMIC-III as stated by Johnson, A.E.W. et al* *(1): The number of structural changes were minimized to achieve the desired level of deidentification and data schema, helping to ensure that MIMIC-III closely represents the raw data collected within the Beth Israel Deaconess Medical Center. Best practice for scientific computing was followed where possible. Code used to build MIMIC-III was version controlled and developed collaboratively within the laboratory. This approach encouraged and facilitated sharing of readable code and documentation, as well as frequent feedback from colleagues. Issue tracking is used to ensure that limitations of the data and code are clearly documented and are dealt with as appropriate. The research community is encouraged to report and address issues as they are found, and a system for releasing minor database updates is in place.*

*For the eICU as stated by Pollard, T.J. et al (4): Data were verified for integrity during the data transfer process from Philips to MIT using MD5 checksums. In order to maintain data fidelity, very little post-processing has been performed. Each participant hospital in the database has customized workflows and clinical documentation processes, and as a result, the reliability and completion of data elements varies on a hospital and/or ICU level. The data archived within eICU-CRD were intended for use during routine clinical care, and not for secondary analysis. Thus, care must be taken when using the data, as inconsistencies which are inconsequential for clinical care may impact analyses performed. A public issue tracker is used as a forum for reporting technical issues and describing solutions.’* (Supplement Page 4; line 69)

4. S2 Table. Body weigth should be vidu height.

**Unfortunately, we do not understand what you mean here.**

5. Sepsis including pneumonia was the most frequent initial diagnosis that increases overtime associated with a lower PaO_2_/FiO_2_. What was the gender Vt difference in this group of patients with probably a lower lung compliance?

**Thank you for this suggestion. We added the results of one posthoc analysis that used only data from patients that had sepsis including pneumonia as the initial diagnosis.**

**We adjusted the Methods, Results, and Discussion** **accordingly, and added a figure to the supplement, as follows:**

*‘Posthoc analyses*

*We performed two posthoc analyses. The first posthoc analysis concerned patients who were admitted because of sepsis, and including patients with pneumo–sepsis. In this analysis we repeated the main analysis to compare temporal shifts in absolute V_T_, V_T, ABW_, and V_T, PBW_ over the calendar years between females and males*.*’* (Page 11; line 267)

and

*‘Posthoc analyses*

*The results of the posthoc analysis concerning 973 female and 1058 male patients with sepsis, did not change the findings, albeit that the differences did not reach statistical significance (****S4 Fig***).(Page 17; line 328)

and

*‘**Fourth, differences between the sexes in the posthoc analysis considering only patients with sepsis were not statistically significant. This, however, was probably to a lack of power since the number of patients used for this analysis was smaller.’* (Page 21; line 420)

and

S4 Fig. Absolute tidal volume and corrected tidal volume (by predicted body weight or absolute body weight) over years and according to tertiles of year in patients with sepsis (Supplement Page 16)

*PBW: predicted body weight; ABW: actual body weight*

*p* values for the sex reflect the overall test for difference between sex over the years while *p* values for the sex x year interaction evaluate if change over time differed by sex.

**Reviewer 2**

1. The authors report an interesting and clinically relevant study in a retrospectively mode on the use of low tidal volume ventilation (LTVV) in female versus male patients over the past years. The main finding was, that the use of LVVT improved over years, but less consequent in women compared to men, but this finiding is more due to the miscalculation of body height than of the gender per se.

**Thank you for your kind words. Concerning the phrase ‘*it is more due to the miscalculation of body height’*, we have to push back a bit: based on our analysis we cannot conclude whether an *incorrect* assessment of body height is responsible for the sex differences. We did discuss this possible explanation in the Discussion. That reads as follows;**

*‘A possible explanation could be a mismeasurement of body height. In the ICU setting, body height is frequently overestimated [17, 18], in particular in shorter patients [13, 19, 20]. In the here analyzed cohorts, males were median 15 cm taller than females, which mirrors previous findings that worldwide males are on average >10 cm taller than females [5, 6, 32]. This difference could explain, at least in part, why females, who are thus usually shorter, are at a higher risk of not receiving LTVV [31, 32]. This is exactly what the mediation analysis suggests––body height is the most important mediator of the sex difference in LTVV.’* (Page 19; line 376)

2. The study is adequately performed in design, statistics and presentation of results. The findings are important and should be recognized by intensivists. I have just a few minor comments:

**Thank you for your kind words, compliments and suggestions, which we are happy to follow.**

3. ABSTRACT - it should read ‘... using the correctly assessed body height for calculation of ideal body weight.’

**See our reply to comment 1**

4. INTRODUCTION – ‘in the past...’ is not correct, since the ARDSNet study is now 21 years old, maybe it could read: ‘Although a landmark study regarding the benefit of LTVV was published in 2000, in the past...’

**Thank you for this suggestion. We changed it accordingly:**

*‘Before a landmark study regarding the benefit of so-called low V_T_ ventilation (LTVV) was published in 2000 [1], it was common practice …’* (Page 5; line 116)

5. INTRODUCTION – misphrase ‘… over recent years over the years ...’

**Thank you, corrected.**

6. REFERENCES - the references are mostly unstructured and not formatted to journals style

**Thank you, corrected.**

7. Since the variation of V_T_ is very dependent from the mode of MV (controlled vs. augmented spontaneously) the authors should add in a sentence which ventilation mode was used in the early phase of MV in the studies.

**Thank you, this is a very valid point. Of note, the eICU database does not contain information with regard to the ventilatory modes. We performed a second posthoc analysis focusing on V_T_ in controlled versus spontaneous ventilation. The findings of this posthoc analysis did not change the findings.**

**We adjusted the Methods, Results, and Discussion** **accordingly, and added a figure to the supplement, as follows:**

*‘**… In* *a second posthoc analysis we explored the differences between sexes during controlled ventilation versus spontaneous ventilation. Also here we followed the main analysis.’* (Page 11; line 271)

and

‘*The second analysis in 1901 patients during controlled ventilation and 1190 patients during assisted ventilation illustrates that differences between the sexes were consistently present during controlled and spontaneous ventilation (****S5*** ***Fig****).*’ (Page 17; line 331)

and

‘*Of note, we only had information on ventilatory mode in the MIMIC-III database, as this was not collected in the eICU study.*’ (Page 21; line 423)

and

S5 Fig. Absolute tidal volume and corrected tidal volume (by predicted body weight or absolute body weight) over years during controlled vs assisted ventilation in the MIMIC-III database (Supplement Page 17)

*PBW: predicted body weight; ABW: actual body weight*

*p* values for the sex reflect the overall test for difference between sex over the years while *p* values for the sex x year interaction evaluate if change over time differed by sex.
